# Plant Growth-Promoting Rhizobacteria Inoculation to Enhance Vegetative Growth, Nitrogen Fixation and Nitrogen Remobilisation of Maize under Greenhouse Conditions

**DOI:** 10.1371/journal.pone.0152478

**Published:** 2016-03-24

**Authors:** Khing Boon Kuan, Radziah Othman, Khairuddin Abdul Rahim, Zulkifli H. Shamsuddin

**Affiliations:** 1 Department of Land Management, Faculty of Agriculture, Universiti Putra Malaysia, Serdang, Selangor, Malaysia; 2 Agrotechnology and Biosciences Division, Malaysian Nuclear Agency, Bangi, Selangor, Malaysia; Estación Experimental del Zaidín (CSIC), SPAIN

## Abstract

Plant growth-promoting rhizobacteria (PGPR) may provide a biological alternative to fix atmospheric N_2_ and delay N remobilisation in maize plant to increase crop yield, based on an understanding that plant-N remobilisation is directly correlated to its plant senescence. Thus, four PGPR strains were selected from a series of bacterial strains isolated from maize roots at two locations in Malaysia. The PGPR strains were screened *in vitro* for their biochemical plant growth-promoting (PGP) abilities and plant growth promotion assays. These strains were identified as *Klebsiella* sp. Br1, *Klebsiella pneumoniae* Fr1, *Bacillus pumilus* S1r1 and *Acinetobacter* sp. S3r2 and a reference strain used was *Bacillus subtilis* UPMB10. All the PGPR strains were tested positive for N_2_ fixation, phosphate solubilisation and auxin production by *in vitro* tests. In a greenhouse experiment with reduced fertiliser-N input (a third of recommended fertiliser-N rate), the N_2_ fixation abilities of PGPR in association with maize were determined by ^15^N isotope dilution technique at two harvests, namely, prior to anthesis (D_50_) and ear harvest (D_65_). The results indicated that dry biomass of top, root and ear, total N content and bacterial colonisations in non-rhizosphere, rhizosphere and endosphere of maize roots were influenced by PGPR inoculation. In particular, the plants inoculated with *B*. *pumilus* S1r1 generally outperformed those with the other treatments. They produced the highest N_2_ fixing capacity of 30.5% (262 mg N_2_ fixed plant^−1^) and 25.5% (304 mg N_2_ fixed plant^−1^) of the total N requirement of maize top at D_50_ and D_65_, respectively. N remobilisation and plant senescence in maize were delayed by PGPR inoculation, which is an indicative of greater grain production. This is indicated by significant interactions between PGPR strains and time of harvests for parameters on N uptake and at. % ^15^N_e_ of tassel. The phenomenon is also supported by the lower N content in tassels of maize treated with PGPR, namely, *B*. *pumilus* S1r1, *K*. *pneumoniae* Fr1, *B*. *subtilis* UPMB10 and *Acinetobacter* sp. S3r2 at D_65_ harvest. This study provides evidence that PGPR inoculation, namely, *B*. *pumilus* S1r1 can biologically fix atmospheric N_2_ and provide an alternative technique, besides plant breeding, to delay N remobilisation in maize plant for higher ear yield (up to 30.9%) with reduced fertiliser-N input.

## Introduction

In Malaysia, both field and sweet corn varieties are highly in demand as animal feed and for human consumption. However, only the latter demand is being widely addressed through maize cultivation as cash crop due to its higher return on investment [1]. In 2012, the total maize production in Malaysia was valued at RM 334.4 million, triple the value of 2008 [2] due to the increasing local and foreign (Brunei and Singapore) demands [1]. Current maize varieties require high rates of fertiliser inputs, particularly fertiliser-N for maximum crop yield and profitability. Fertiliser-N is also required to replenish the N harvested in stover and ear of the previous season, which can be as high as 115 kg N ha^−1^ [3]. Thus, many farmers practise ‘insurance’ application of fertiliser-N to ensure adequate N supply for crop growth. This practice demands high amounts of fertilisers to grow maize, although only 30–50% of the fertiliser-N applied is absorbed by plants [4], the rest are either rendered unavailable as adsorbed soil organic-N or leached into the environment.

Plant growth-promoting rhizobacteria (PGPR) isolated as free-living soil bacteria from plant rhizosphere can decrease chemical fertiliser-N use and increase plant growth and yield when associated with plant roots and other plant parts [5]. Several bacteria such as *Azospirillum* [6], *Klebsiella* [7], *Burkholderia* [8], *Bacillus* [9] and *Pseudomonas* [10] have been identified as PGPR to maize plant through biological nitrogen fixation (BNF), phosphate solubilisation, phytohormone production (e.g., auxin, gibberellin and cytokinin) and biological control of soil pathogens. BNF by PGPR has been reported to contribute up to 12–70% of total N uptake in field crops or 26.7 kg N ha^−1^ (70% of total N uptake) in maize [6], sugarcane [11] and oil palm [9]. Generally, it has been estimated that up to 65% of N used in agriculture is contributed by BNF, and that it will be an increasingly important component in future plant-N management [12]. In addition, N remobilisation in plant plays a crucial role to reutilise the N from vegetative plant parts for developing organs, especially seeds/grains. N remobilisation occurs naturally throughout plant growth and whenever the plant requires it, although the onset of leaf senescence is identified as the main stimulus [13]. It was reported that 50–90% of N in wheat and maize grains is remobilised from the leaf-N [14].

However, present information on indigenous PGPR associations with maize plant towards BNF and their influence on N remobilisation is still limited. Thus, a concerted effort is needed for an effective plant-N management. Therefore, the aim of this study was to select effective PGPR strains from a series of indigenous bacterial strains by biochemical characterisations and plant growth promotion assays. These selected strains and a reference strain, UPMB10 were identified using 16S rDNA gene analysis and further inoculated to maize plants grown under greenhouse conditions to estimate the amount of N_2_ fixed and their influence on plant-N remobilisation prior to anthesis and ear harvest, using the ^15^N isotope dilution technique.

## Materials and Methods

### Bacterial isolation

Bacterial strains were isolated from roots of healthy maize plants grown at University Agricultural Park, Universiti Putra Malaysia (UPM), Selangor (2°58’52.17” N, 101°42’44.94” E) and Lentang Village in Sik, Kedah (6°2’57.84” N, 100°50’24.60” E) using the modified method of Hoben and Somasegaran [15]. Three fresh root tips (3 cm) with sufficient adhered rhizosphere soils were collected in McCartney bottles which contained 10 mL of sterilised distilled water. The bottle was shaken for 30 s with a vortex mixer at 1000 rpm and serially diluted with ten-fold dilutions prior to spreading the root suspension on tryptic soy agar medium (TSA; Merck KGaA, Germany) to isolate the rhizospheric bacteria. The same roots were surface-sterilised with 70% ethanol for 5 min, followed by 1% of sodium hypochlorite for 30 s and washed five times with sterilised distilled water. The roots were streaked on TSA plates to check the sterilisation efficiency and aseptically smashed with mortar and pestle to isolate the endophytic bacteria on TSA medium. The TSA plates were invertedly incubated for 24 h at 30±2°C. Colonies with visual morphological differences were selected and sub-cultured to obtain pure colonies. A total of 57 bacterial strains were isolated and screened for N_2_-fixing activity, phosphate solubilisation, indole-3-acetic acid (IAA) production and plant growth promotion assay. A parallel experiment on plant growth promotion assays indicated that PGPR inoculations with Fr1, S1r1 and S3r2 produced the highest maize plant top biomass and N uptake (S1 and S2 Figs). Thus, these three PGPR strains and a negative and reference strain, Br1 and UPMB10, respectively, were subsequently selected for the pot experiment. The UPMB10 strain was isolated from oil palm root [16] and is used in the commercial product Bacto-10^™^. Maize and oil palm being monocotyledonous plants form a monophyletic group that shares similar arrangements of vascular bundles in the stem, parallel major leaf veins and adventitious root system.

### Biochemical characterisation

Initial screening for N_2_-fixing activity of the pure bacterial cultures was determined on N-free semi-solid malate medium (Nfb) [17]. The plates were incubated for 24 h at 30±2°C, whereby a colour change from pale green to blue would qualitatively indicate the positive effect of N_2_-fixing activity. Phosphate solubilisation test was conducted according to the method of Pikovskaya [18], where the presence of clear halo zones around the colonies would indicate positive phosphate solubilisation. IAA production was determined according to the modified method of Glickmann and Dessaux [19]. PGPR cultures were grown in tryptic soy broth (TSB) which contained 2 mg mL^−1^ of L-tryptophan and incubated on a rotary shaker at 200 rpm under room temperature, 28±2°C for 24 h. The cultures were centrifuged at 7000 rpm for 7 min and the supernatants were quantified spectrophotometrically at 535 nm with 2 mL of Salkowski’s reagent (2% of 0.5 M FeCl_3_ in 35% perchloric acid) after 25 min. The IAA concentrations were estimated from a standard IAA curve.

### 16S rDNA gene analysis of selected bacterial strains

Total genomic DNA was extracted using Genomic DNA Mini Kit (Yeastern Biotech Co. Ltd.) according to the supplier’s instructions and used as DNA template in polymerase chain reaction (PCR) for amplification of the 16S rDNA gene. The DNA purity was quantified at 260 nm and 280 nm using NanoDrop Spectrophotometer (ND1000, Thermo Fisher Scientific Inc.), 1.6 to 2.2, to detect protein contamination in the DNA [20]. PCR amplification was performed in a reaction mixture containing 50 ng genomic DNA template, 1X reaction buffer, 200 μM dNTPs mixture, 1.5 mM MgCl_2_, 2.5 U *Taq* DNA polymerase (Thermo Fisher Scientific), 0.16 μM of each primer 27F (5’-AGAGTTTGATCTTGGCTCAG-3’) and 1492R (5’-TACGGTTACCTTGTTACGACTT-3’) [21], and ultra-pure sterilised water to a final volume of 50 μL. These primers allowed an approximate 1500 bp of DNA fragments to be amplified. The DNA amplification was performed in a thermal cycler (MJ Mini Personal Thermal Cycler PTC-1148, Bio-Rad) by an initial denaturation at 94°C for 5 min, followed by 30 amplification cycles of denaturation at 94°C for 30 s, annealing at 55°C for 30 s, extension at 72°C for 1 min and a final extension step at 72°C for 5 min. The suitability of DNA amplification was visualised by electrophoresis of PCR products with 6X loading dye (Thermo Scientific^™^) at 5:1 (PCR product: dye) ratio and a marker (1kb DNA ladder, Fermentas GeneRuler^™^) in 1% (w/v) agarose gel in 1X Tris-acetate EDTA (TAE) buffer for 1 h at 80 V. The agarose gel was stained with GelRed^™^ (Biotium Inc.) for 40 min and examined under UV light in a UV transilluminator (Bio-Rad Molecular Imager^®^ Gel Doc^™^ XR+ System). Gel image was captured with Image Lab software (Version 4.1, Bio-Rad Laboratories) (S3 Fig). DNA fragments were extracted from the gel by slicing the target bands under UV light and purified using QIAquick gel extraction kit (Qiagen^®^). The purified DNA products were sent for sequencing by First BASE Laboratories Sdn. Bhd. (www.base-asia.com) using BigDye^®^ Terminator 3.1 cycle sequencing kit (Applied BioSystems). The quality of 16S rDNA sequences was checked manually by using Applied Biosystems Sequence Scanner Software (version 2.0). The sequences were analysed using the BLAST Sequence Similarity Search to identify the most closely related members in the NCBI GenBank DNA database (www.ncbi.nlm.nih.gov/geo). The partial 16S rDNA sequences of the PGPR strains were submitted to the NCBI database under their respective accession number as follows: *Bacillus subtilis* UPMB10 (KP641618), *Klebsiella* sp. Br1 (KP257586), *Klebsiella pneumoniae* Fr1 (KP641617), *Bacillus pumilus* S1r1 (KP295962) and *Acinetobacter* sp. S3r2 (KP295963).

### Phylogenetic analysis

The partial 16S rDNA sequences of UPMB10, Br1, Fr1, S1r1 and S3r2 were aligned with the most closely related bacteria sequences obtained from the NCBI database using MUSCLE [22]. The tree was constructed with Mega version 5 software package [23] by using the maximum likelihood method from distance calculated by the method of Kimura two-parameter model with a discrete Gamma distribution [24]. Gaps were treated by partial deletion and bootstrap analysis was done by using 2000 replications. *Streptomyces griseus* FGQ9 (HQ202539) was used as an outgroup sequence.

### Pot experiment

The experimental soil (Serdang series soil, Typic Paleudult) was collected from 0–15 cm soil depth at the University Agriculture Park, UPM (2°59'12.5" N, 101°38'52.9" E). The soil was air-dried, ground, sieved (2 mm mesh) and analysed for its physico-chemical properties. The soil had pH 5.1 (1:2.5, soil:water ratio; MeterLab^®^ PHM210); total N (semi-micro Kjeldahl method) [25], 0.46%; total C (Leco CR-12 carbon analyser) [26], 3.2%; available P (Bray I) [27], 31.6 mg kg^−1^; exchangeable K (NH_4_OAc) [28], 46.8 mg kg^−1^ and field capacity (pressure plate method) [29], 26.8%. Exactly 20 kg of the soil was weighed and placed in each undrained polybag (60 cm, D × 50 cm, H). Uniformed doses of Christmas Island Rock Phosphate (CIRP, 30% P_2_O_5_) and Muriate of Potash (MOP, 60% K_2_O) at the recommended rates of 60 kg P_2_O_5_ ha^−1^ and 40 kg K_2_O ha^−1^ equivalents [30], respectively, were applied to each polybag. The ^15^N-labelled urea (46% N, 4.72 at. % ^15^N_e_) at 40 kg N ha^−1^ equivalent (a third of recommended fertiliser-N rate) [30] was dissolved in 100 mL distilled water and applied to each polybag as a tracer. The experiment was conducted in a randomised complete block design, with two harvests at 50 and 65 days after planting (DAP; D_50_ and D_65_) with four blocks and 0.75 m × 0.25 m planting distance between the polybags. The six treatments imposed were: (i) killed *B*. *subtilis* UPMB10 inoculum, autoclaved at 121°C for 20 min (Uninoculated control), (ii) *B*. *subtilis* UPMB10 inoculation (Reference control), (iii) *Klebsiella* sp. Br1 inoculation (Negative control), (iv) *K*. *pneumoniae* Fr1 inoculation, (v) *B*. *pumilus* S1r1 inoculation and (vi) *Acinetobacter* sp. S3r2 inoculation. Six maize seeds of similar size and shape, var. Hibrimas, were sowed and thinned to two per pot at 7^th^ DAP (D_7_). The PGPR inocula were grown in TSB medium for 24 h (200 rpm, 26±2°C) and cell suspensions were adjusted to OD_600_ between 1.4 and 2.0, which corresponded to the total plate counts of ca. 10^9^ cfu mL^−1^, as determined on the TSA medium. Each polybag was inoculated with 20 mL of respective inocula (live or killed) on D_0_, D_7_ and D_35_. The polybags were watered and weighted daily to maintain at field capacity throughout the experiment to avoid any possible loss of applied ^15^N labelled fertiliser through denitrification, if the soil contained excess moisture. The total bacterial colonisations in soil, rhizosphere and root-endosphere were determined according to the method of Hoben and Somasegaran [15] from the collected soil and fresh root samples at D_65_ harvest.

### ^15^N abundance plant analysis

At harvest (D_50_ and D_65_), each maize plant was separated into roots, young, ear and old leaves, stalk, tassel and ear (D_65_ only). The ear bearing leaf and its immediate lower leaf were labelled as ear leaves. The upward and downward leaves from the ear leaves were respectively labelled as young and old leaves. Every component was weighed, oven-dried (65±2°C, 72 h) and ground (<1 mm) to determine the plant tissue N (except root) using the semi-micro Kjeldahl method [25] and ^15^N abundance using an emission spectrometer (NOI7, Fisher Germany) at Malaysian Nuclear Agency in Bangi, Selangor. The ^15^N abundance in the sample was corrected for the natural ^15^N abundance present in the environment (0.3663 at. % ^15^N_e_). Meanwhile, the N_2_ fixation in the whole maize plant (plant top basis) was calculated from the mean weighted atom excess (WAE) using the following formula [31]:

$$\text{WAE}=\frac{\text{AEa }\times \text{ TNa }+\text{ AEb }\times \text{ TNb }+\text{ AEc }\times \text{ TNc }+\text{ AEd }\times \text{ TNd }+\text{ AEe }\times \text{ TNe }+\text{ AEf }\times \text{ TNf}}{\text{TNa }+\text{ TNb }+\text{TNc }+\text{ TNd }+\text{TNe }+\text{ TNf}}$$

Where AE = at. % ^15^N_e_, TN = total N of a, b, c, d, e and f = tassel, young, ear and old leaves, stalk and ear, respectively, ear was only available in D_65_ harvest. The proportions of N derived from the atmosphere (% Ndfa) was calculated, as follows:

$$\text{Ndfa }\left(\%\right)=\left(1-\frac{\text{at}.\;\%{\;}^{15}{\text{N}}_{\text{e}}\text{ fixing plant}}{\text{at}.\;\%{\;}^{15}{\text{N}}_{\text{e}}\text{ non-fixing plant}}\right)\times 100$$

### Statistical analysis

Data were analysed using one-way analysis of variance procedure (ANOVA) followed by Duncan’s Multiple Range Test (DMRT) at p<0.05 using Statistical Analysis System software (SAS version 9.0) [32]. Pearson correlation of coefficient test was performed to estimate the relationships between related variables.

## Results

### PGPR strain characterisation

The plant growth-promoting (PGP) abilities of the four selected strains and a reference strain, UPMB10 are shown in Table 1. These five strains gave positive reactions on Nfb media. In particular, four strains (UPMB10, Br1, S1r1 and S3r2) showed the clearest halo zones around their bacterial colonies grown on the Pikovskaya media, indicating higher phosphate solubilisation abilities compared to Fr1 strain. Treatment with Fr1 strain, however, produced the significantly highest IAA at 13 μg mL^−1^ compared to the other strains (5–11 μg mL^−1^).

**Table 1:** Plant growth promoting (PGP) abilities of the five strains used in the glasshouse experiment.

Strain	Nfb reaction	Phosphate solubilisation ^a^	IAA ^b^ (μg mL^−1^)
**UPMB10**	+	+++	10.10 b
**Br1**	+	+++	4.91 c
**Fr1**	+	++	12.99 a
**S1r1**	+	+++	10.55 b
**S3r2**	+	+++	10.70 b

^a^ Phosphate solubilisation strength;

^b^ Values (means of three replicates) not sharing a common letter differ significantly (P<0.05) from each other (DMRT).

### Molecular identification of PGPR strains

**Fig 1:**
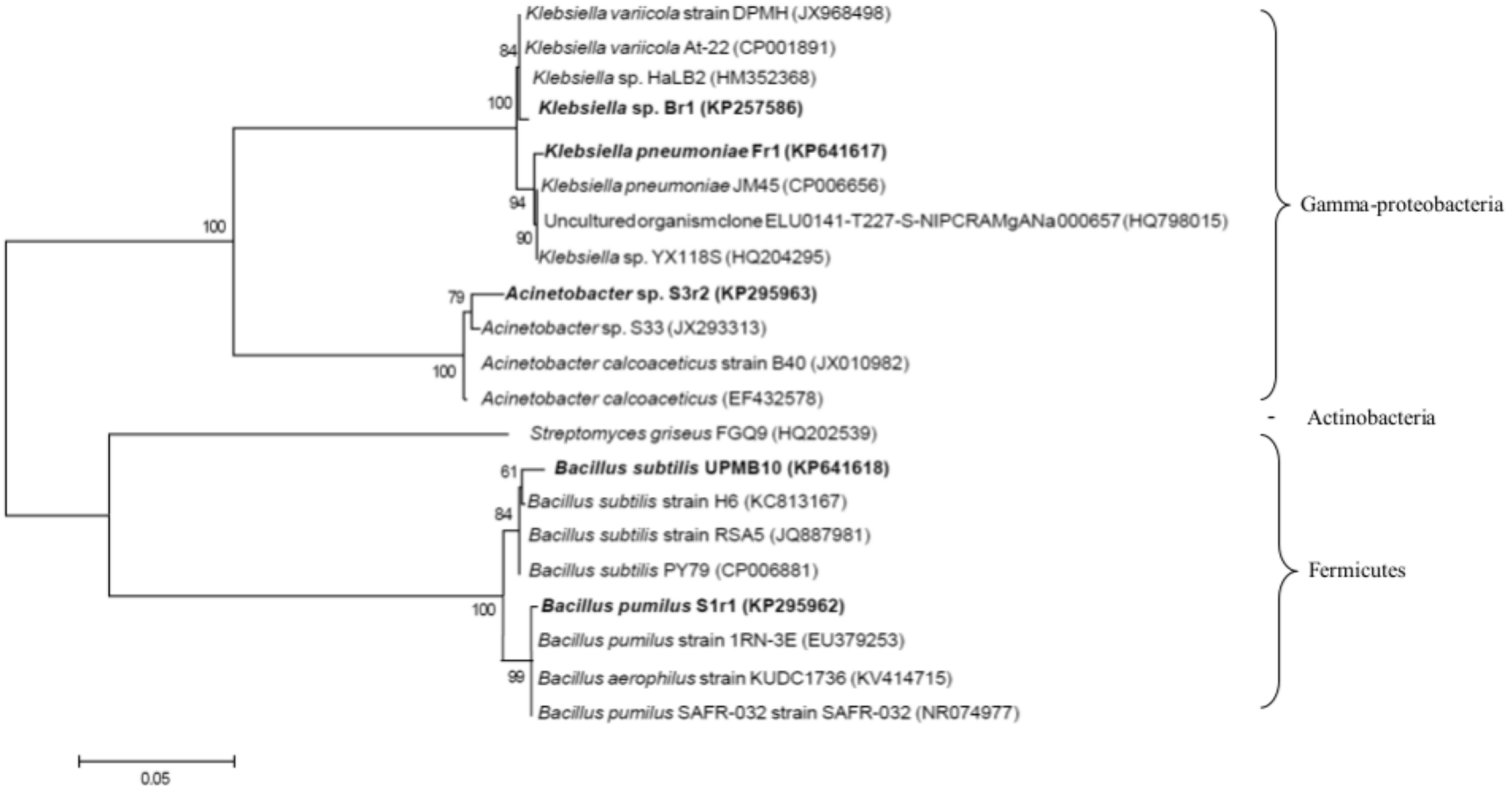
Phylogenetic tree derived from analysis of the partial 16S rDNA sequences of UPMB10, Br1, Fr1, S1r1, S3r2 and related sequences obtained from NCBI database. *Streptomyces griseus* FGQ9 (HQ202539) belonging to the *Streptomyces* genus is used as an outgroup sequence. Scale bar, 0.05 substitutions per nucleotide position.

Analysis of 16S rDNA sequences from the NCBI database suggested that UPMB10, Br1, Fr1, S1r1 and S3r2 were most closely related to *Bacillus subtilis* (98% similarity), *Klebsiella* sp. (99% similarity), *Klebsiella pneumoniae* (99% similarity), *Bacillus pumilus* (99% similarity) and *Acinetobacter* sp. (98% similarity), respectively. A phenogram showing the genetic relationship among the PGPR strains and their most closely related bacteria obtained from NCBI database is presented in Fig 1. The PGPR strains were indicated to belong to two subdivisions and three different genera of bacteria: (i) *Gamma-proteobacteria*: *Klebsiella* spp. (Br1 and Fr1) and *Acinetobacter* spp. (S3r2); (ii) *Firmicutes*: *Bacillus* spp. (UPMB10 and S1r1).

### Total bacterial colonisation

PGPR inoculations increased rhizobacterial colonisation around the maize roots and their surrounding soils compared to uninoculated control at ear harvest (D_65_) (Table 2). Rhizosphere had the highest total bacterial population, followed by soil and root-endosphere. Inoculation with *Klebsiella* sp. Br1 and *Acinetobacter* sp. S3r2 gave significantly higher bacterial populations in their respective soils, with around 250–300% over the uninoculated control (1.98 x 10^7^ cfu g^−1^). In the root-endosphere, the PGPR inoculations particularly with *Acinetobacter* sp. S3r2 significantly stimulated up to 757 folds increase in total bacterial population compared to the uninoculated control (96 cfu cm^−1^ fresh root).

**Table 2:** Total bacterial population at ear harvest (D_65_).

Treatments ^a^	Soil (10^7^ cfu g^−1^ dry soil)	Rhizosphere (10^8^ cfu g^−1^ root dry weight)	Root-endosphere (10^2^ cfu cm^−1^ fresh root)
**Uninoculated control**	1.98±0.06 b	1.72±0.15 a	0.96±0.03 b
***Bacillus subtilis* UPMB10**	2.41±0.22 b	2.97±0.97 a	5.90±0.75 b
***Klebsiella* sp. Br1**	5.85±1.61 a	6.25±1.95 a	3.46±0.75 b
***Klebsiella pneumoniae* Fr1**	2.36±0.18 b	4.84±1.16 a	10.90±0.72 b
***Bacillus pumilus* S1r1**	4.22±0.96 ab	2.98±0.36 a	3.34±0.19 b
***Acinetobacter* sp. S3r2**	5.39±1.12 a	3.79±1.08 a	726.77±89.39 a

^a^ For each response variable, values (means of four replicates) not sharing a common letter differ significantly (P<0.05) from each other (DMRT).

### Plant biomass yield

Generally, inoculation with PGPR significantly increased the dry biomass of whole plant and various plant parts of maize prior to anthesis (D_50_) and ear harvest (D_65_), except in tassel (D_65_), young leaves (D_65_), old leaves (D_50_ and D_65_), stalk (D_50_) and root (D_65_) (Table 3). Among the inoculated treatments, the inoculation with *B*. *pumilus* S1r1 gave significantly highest biomass in tassel (D_50_), young leaves (D_50_), ear leaves (D_65_) and ear. Comparatively, the inoculation with *K*. *pneumoniae* Fr1 gave similar biomass in tassel (D_50_), young leaves (D_50_), ear leaves (D_50_ and D_65_), stalk (D_65_), ear (D_65_) and root (D_50_). Both inoculations with *B*. *pumilus* S1r1 (42.3 g, 68.0 g) and *K*. *pneumoniae* Fr1 (45.2 g, 67.2 g) gave significantly higher total plant biomass compared to uninoculated control (33.6 g, 54.2 g) at D_50_ and D_65_ harvests. Plant stalk accounted for the highest proportion of dry biomass, making up to 40.6% and 38.9% of total plant biomass at D_50_ and D_65_, respectively. In D_50_, this was followed by tassel (15.2%), ear leaves (13.9%), young leaves (12.3%), older leaves (11.8%) and roots (6.1%). In D_65_, this was followed by ear (16.5%), young leaves (10.3%), ear leaves (9.1%), tassel (9.1%), old leaves (8.2%) and roots (7.8%). The part of maize plant that experienced a significantly lower biomass at D_65_ was the tassel (5.4–6.5 g to 5.4–5.7 g). On the contrary, the plant parts that experienced significant increment of biomass at D_65_ were the young leaves (4.2–5.7 g to 5.5–7.3 g), stalk (13.9–18.0 g to 21.7–26.1 g) and roots (1.9–2.7 g to 4.3–5.3 g).

**Table 3:** Plant biomass yield in the whole plant and in the different parts of maize plant inoculated with PGPR strains at D_50_ (before anthesis) and D_65_ (ear harvest).

PGPR strains ^a^	Dry biomass yield (g plant^−1^), Mean±SEM															
	Tassel		Young leaves		Ear leaves		Old leaves		Stalk		Ear		Root		Whole plant	
	D_50_	D_65_	D_50_	D_65_	D_50_	D_65_	D_50_	D_65_	D_50_	D_65_	D_50_	D_65_	D_50_	D_65_	D_50_	D_65_
**Control**	5.43±0.45 bA	5.38±0.36 aA	4.20±0.35 bB	5.45±0.36 aA	4.53±0.67 cA	4.39±0.46 cA	3.71±0.50 aA	4.20±1.06 aA	13.87±0.82 aB	21.85±0.32 bA	N/A	8.65±0.71 c	1.91±0.13 cB	4.29±0.22 aA	33.64±2.14 dB	54.20±2.85 bA
**UPMB10**	6.05±0.15 abA	5.46±0.19 aB	4.29±0.59 bA	5.74±0.62 aA	5.13±0.55 bcA	5.60±0.30 abA	4.08±0.70 aA	4.38±0.33 aA	15.90±0.73 aB	22.89±1.70 abA	N/A	9.78±0.43 b	2.48±0.10 abB	4.74±0.45 aA	37.92±1.39 cB	58.58±2.01 bA
**Br1**	5.61±0.28 bA	5.36±0.38 aA	4.30±0.57 bA	5.62±0.86 aA	5.59±0.86 abA	4.79±0.36 bcA	4.49±0.64 aA	4.59±0.62 aA	15.87±1.05 aB	21.67±0.96 bA	N/A	9.41±0.59 bc	2.20±0.12 bcA	4.35±0.72 aA	38.05±2.18 cB	55.78±3.38 bA
**Fr1**	6.36±0.13 aA	5.66±0.29 aB	5.64±0.76 aA	6.99±0.40 aA	6.42±0.31 aA	6.61±0.43 aA	6.09±0.23 aA	5.84±0.12 aA	17.97±0.72 aB	26.10±0.52 aA	N/A	11.21±0.23 a	2.74±0.30 aB	4.80±0.62 aA	45.21±0.74 aB	67.20±2.00 aA
**S1r1**	6.51±0.29 aA	5.56±0.36 aB	5.69±0.39 aB	7.31±0.21 aA	5.85±0.83 abA	6.63±0.55 aA	5.17±0.37 aA	6.18±0.16 aA	16.54±0.61 aB	25.68±0.68 aA	N/A	11.32±0.39 a	2.51±0.05 abB	5.30±0.48 aA	42.28±1.06 abB	67.98±2.38 aA
**S3r2**	6.29±0.44 aA	5.56±0.34 aB	5.17±0.48 abA	6.16±0.93 aA	5.61±0.51 abA	5.05±0.46 bcA	4.50±0.32 aA	4.66±0.42 aA	16.34±0.27 aB	22.98±1.65 abA	N/A	9.52±0.45 bc	2.62±0.20 abB	4.79±0.63 aA	40.53±1.62 bcB	58.72±3.88 bA
**Mean by harvests**	6.04±0.14 A	5.50±0.12 B	4.88±0.24 B	6.21±0.27 A	5.52±0.27 A	5.51±0.24 A	4.67±0.24 A	4.97±0.25 A	16.08±0.37 B	23.52±0.54 A	N/A	9.98±0.27	2.41±0.09 B	4.72±0.21 A	39.61±0.96 B	60.41±1.51 A

^a^ For each response variable, values (means of four replicates) not sharing a common letter, lower case (e.g. a, b) in the vertical columns for each plant part within every harvest and upper case (e.g. A, B) in the horizontal lines for each plant part between harvests (D_50_, D_65_), differ significantly (P<0.05) from each other (DMRT).

N/A = Not available before anthesis.

### Total nitrogen uptake

Inoculation with PGPR significantly increased the N uptake in all plant parts and plant tops of maize at D_50_ and D_65_ harvests, except in the tassel (D_65_) (Table 4). Meanwhile, inoculation with *B*. *pumilus* S1r1 significantly increased the total N uptake in plant top by 55.1% and 50.1%, followed by *K*. *pneumoniae* Fr1 with similar increments of 61.4% and 48.4% at D_50_ and D_65_, respectively, in comparison to uninoculated control. *B*. *pumilus* S1r1 and *K*. *pneumoniae* Fr1 also significantly increased the N uptake in tassel (D_50_ only), young, ear and old leaves, stalk and ear (D_65_) of maize. Generally, the significant differences in the N uptake of plant parts between D_50_ and D_65_ harvests were shown as a decrease in total N in the tassel and increased total N in the young leaves (S1r1 only), stalk and plant top.

**Table 4:** Distribution of total N uptake (mg plant^−1^) in the plant top and in the different parts of maize plant inoculated with PGPR strains at D_50_ (before anthesis) and D_65_ (ear harvest).

PGPR strains ^a^	Total N uptake (mg plant^−1^), Mean±SEM													
	Tassel		Young leaves		Ear leaves		Old leaves		Stalk		Ear		Plant top	
	D_50_	D_65_	D_50_	D_65_	D_50_	D_65_	D_50_	D_65_	D_50_	D_65_	D_50_	D_65_	D_50_	D_65_
**Control**	121±14 cA	110±8 aA	104±10 bA	126±11 bA	104±14 dA	97±11 cA	81±10 cA	88±22 bA	206±10 bB	317±5 bA	N/A	142±10 d	617±36 dB	880±52 dA
**UPMB10**	161±5 bA	123±5 aB	121±16 bA	179±20 abA	135±15 cA	149±7 abA	110±16 bcA	117±10 bA	250±11 aB	336±32 bA	N/A	191±15 bc	776±37 cB	1095±55 bA
**Br1**	138±5 cA	114±12 aA	120±12 bA	160±25 bA	150±22 bcA	122±10 bcA	122±19 bcA	121±18 bA	248±12 aB	310±8 bA	N/A	168±9 c	776±38 cB	994±45 cA
**Fr1**	179±6 abA	126±8 aB	172±21 aA	223±16 aA	185±9 aA	180±12 aA	173±8 aA	166±4 aA	286±13 aB	400±15 aA	N/A	212±11 ab	996±26 aB	1306±39 aA
**S1r1**	186±12 aA	124±11 aB	176±11 aB	234±6 aA	169±24 abA	182±18 aA	147±13 abA	168±3 aA	280±15 aB	391±12 aA	N/A	223±8 a	957±38 aB	1321±38 aA
**S3r2**	173±13 abA	121±12 aB	159±14 aA	180±28 abA	156±14 bcA	127±15 bcA	119±7 bcA	124±10 bA	247±6 aB	328±23 bA	N/A	173±9 c	854±44 bB	1052±75 bcA
**Mean by harvests**	160±6 A	119±4 B	142±8 B	184±10 A	150±8 A	143±8 A	125±8 A	131±8 A	253±7 B	347±10 A	N/A	185±7	829±29 B	1108±38 A

^a^ For each response variable, values (means of four replicates) not sharing a common letter, lower case (e.g. a, b) in the vertical columns for each plant part within every harvest and upper case (e.g. A, B) in the horizontal lines for each plant part between harvests (D_50_, D_65_), differ significantly (P<0.05) from each other (DMRT).

N/A = Not available before anthesis.

In the tassel, the mean N uptake at D_65_ (119 mg plant^−1^) was significantly lower compared to D_50_ (160 mg plant^−1^), as shown among the inoculated treatments (except for *Klebsiella* sp. Br1). In young leaves, the mean N uptake was significantly higher at D_65_ (184 mg plant^−1^) compared to D_50_ (142 mg plant^−1^). However, this effect was evident only in inoculated treatment with *B*. *pumilus* S1r1. Similarly, the mean N uptake in the stalk and plant top significantly increased at D_65_ (347; 1108 mg plant^−1^) compared to D_50_ (253; 829 mg plant^−1^). The highest accumulation of N was found in plant stalk, containing 30.5% and 31.3% of the total plant top N at D_50_ and D_65_, respectively. The lowest accumulation of N was found in old leaves (D_50_) and plant tassel (D_65_) at 15.1% and 10.7% of the total plant top N, respectively. In general, majority of total N (70–81%) in the plant top of maize was accumulated before anthesis (D_50_).

### PGPR biological nitrogen fixation and plant nitrogen remobilisation

Maize inoculated with PGPR generally had significantly lower weighted ^15^N atom excess (at. % ^15^N_e_) in the plant top and in the different parts of maize than the uninoculated control at D_50_ and D_65_ harvests (Table 5). Among the inoculated treatments, inoculation with *B*. *pumilus* S1r1 was significantly lowest at. % ^15^N_e_ in all the plant parts at D_50_ and D_65_. Similarly, inoculation with *K*. *pneumoniae* Fr1 also recorded significantly lower at. % ^15^N_e_ in the young leaves, ear leaves (D_65_ only), old leaves and stalk. In general, the plant tops and all parts of maize (except for plant stalk and ear) experienced significantly lower mean at. % ^15^N_e_ value at D_65_ than D_50_. However, the lower at. % ^15^N_e_ value in tassel at D_65_ among the inoculated maize plants was not statistically significant, except for the *Klebsiella* sp. Br1 treatment. Among the various plant parts, the lowest and highest concentrations of at. % ^15^N_e_ were found in the ear (0.651–0.885 at. % ^15^N_e_) and old leaves (1.035–1.604 at. % ^15^N_e_), respectively.

**Table 5:** Distribution of %^15^N atom excess (at. % ^15^N_e_) in the different plant parts and the mean weighted atom excess (WAE) for the plant top of maize inoculated with PGPR strains at D_50_ (before anthesis) and D_65_ (ear harvest).

PGPR strains ^a^	%^15^N atom excess (Mean±SEM)													
	Tassel		Young leaves		Ear leaves		Old leaves		Stalk		Ear		Plant top (WAE)	
	D_50_	D_65_	D_50_	D_65_	D_50_	D_65_	D_50_	D_65_	D_50_	D_65_	D_50_	D_65_	D_50_	D_65_
**Control**	1.078±0.020 aA	0.941±0.008 aB	1.190±0.021 aA	1.045±0.023 aB	1.273±0.042 aA	1.133±0.048 aB	1.604±0.052 aA	1.474±0.028 aA	1.116±0.019 aA	1.092±0.042 aA	N/A	0.885±0.016 a	1.259±0.013 aA	1.104±0.003 aB
**UPMB10**	0.876±0.014 cA	0.895±0.015 abcA	0.845±0.025 dA	0.847±0.017 cA	0.974±0.014 cA	0.702±0.014 cB	1.270±0.026 cA	1.204±0.023 cB	0.960±0.021 bA	0.893±0.018 bB	N/A	0.739±0.018 c	0.986±0.009 cA	0.885±0.003 cB
**Br1**	0.955±0.013 bA	0.910±0.016 abB	0.964±0.036 cA	1.003±0.029 abA	1.028±0.030 bcA	0.887±0.014 bB	1.432±0.049 bA	1.263±0.045 bcB	0.955±0.029 bA	0.940±0.020 bA	N/A	0.826±0.019 b	1.080±0.012 bA	0.987±0.008 bB
**Fr1**	0.877±0.011 cA	0.873±0.016 bcA	0.840±0.024 dA	0.836±0.018 cA	0.890±0.013 dA	0.728±0.032 cB	1.081±0.033 dA	1.057±0.036 dA	0.923±0.039 bA	0.900±0.016 bA	N/A	0.695±0.020 d	0.921±0.005 dA	0.854±0.014 dB
**S1r1**	0.815±0.010 dA	0.845±0.030 cA	0.808±0.030 dA	0.813±0.014 cA	0.797±0.014 eA	0.665±0.020 cB	1.056±0.037 dA	1.035±0.006 dA	0.924±0.032 bA	0.934±0.041 bA	N/A	0.651±0.015 e	0.875±0.009 eA	0.823±0.008 eB
**S3r2**	0.970±0.029 bA	0.896±0.011 abcA	1.071±0.032 bA	0.965±0.007 bB	1.105±0.022 bA	0.950±0.023 bB	1.373±0.033 bcA	1.352±0.043 bA	0.974±0.021 bA	0.951±0.036 bA	N/A	0.830±0.021 b	1.108±0.009 bA	1.009±0.010 bB
**Mean by harvests**	0.928±0.019 A	0.893±0.009 B	0.953±0.031 A	0.918±0.020 B	1.011±0.033 A	0.844±0.036 B	1.303±0.043 A	1.231±0.034 B	0.975±0.017 A	0.952±0.018 A	N/A	0.771±0.018	1.038±0.027 A	0.943±0.021 B

^a^ For each response variable, values (means of four replicates) not sharing a common letter, lower case (e.g. a,b) in the vertical columns for each plant part within every harvest and upper case (e.g. A,B) in the horizontal lines for each plant part between harvests (D_50_, D_65_), differ significantly (P<0.05) from each other (DMRT).

N/A = Not available before anthesis.

Maize inoculated with PGPR gave marked increases in % Ndfa and amounts of N_2_ fixed in the plant top and different plant parts of maize at D_50_ and D_65_ harvests, except for the plant stalk (Table 6). In the maize plant top, inoculation with *B*. *pumilus* S1r1 gave 30.5% Ndfa (262 mg N fixed plant^−1^) and 25.5% Ndfa (304 mg N fixed plant^−1^) at D_50_ and D_65_, respectively. Generally, the mean % Ndfa in the inoculated maize plant top at D_65_ (17.5%) was significantly reduced in comparison to D_50_ (21.1%), despite the significant increase in the mean amount of fixed N by 12.9%. Based on the fixed N content of the inoculated maize plants, the estimated mean N_2_ fixation rate was higher prior to anthesis (D_50_) at 3.5 mg N fixed plant^−1^ day^−1^ compared to after anthesis (D_65_) at 3.1 mg N fixed plant^−1^ day^−1^.

**Table 6:** Estimates of proportions of N_2_ derived from atmosphere (% Ndfa) and amounts of N_2_ fixed (in parenthesis, mg N fixed plant^−1^) in the plant top and different plant parts of maize inoculated with PGPR strains at D_50_ (before anthesis) and D_65_ (ear harvest).

PGPR strains ^a^	% Ndfa (amount of N_2_ fixed, mg plant^−1^), Mean±SEM													
	Tassel		Young leaves		Ear leaves		Old leaves		Stalk		Ear		Plant top	
	D_50_	D_65_	D_50_	D_65_	D_50_	D_65_	D_50_	D_65_	D_50_	D_65_	D_50_	D_65_	D_50_	D_65_
**UPMB10**	18.7±0.9 bA (30.3±2.2 bA)	4.9±0.8 aB (6.3±1.0 aB)	28.9±3.3 aA (35.5±7.2 bA)	18.9±1.3 aA (33.5±3.4 bA)	23.3±1.5 cB (31.0±2.7 bB)	37.8±2.3 aA (56.0±3.7 aA)	20.7±2.1 bA (22.8±4.7 bA)	18.2±2.8 bA (21.8±4.3 bA)	13.9±1.0 aA (35.3±4.1 aA)	18.0±2.5 aA (58.3±5.6 aA)	N/A	16.5±1.2 c (31.3±2.2 c)	21.7±0.3 cA (154.5±10.1 bB)	19.9±0.5 bB (206.8±4.3 bA)
**Br1**	11.4±2.0 cA (15.8±2.8 cA)	3.3±1.2 aB (4.0±1.7 aB)	19.1±2.0 bA (22.8±3.4 bcA)	4.1±1.1 bB (6.0±1.0 cB)	18.8±4.7 cA (27.0±5.9 bA)	21.3±3.7 bA (25.3±3.5 bA)	10.7±1.9 cA (13.0±3.2 bA)	14.3±2.3 bcA (16.8±2.5 bcA)	14.5±1.1 aA (35.8±2.9 aA)	13.4±4.9 aA (40.5±14.0 aA)	N/A	6.7±0.8 d (11.5±1.8 d)	14.2±1.7 dA (114.5±15.7 cA)	10.6±0.8 cA (103.8±9.5 cA)
**Fr1**	18.5±2.1 bA (33.0±3.9 bA)	7.2±1.9 aB (8.8±2.2 aB)	29.4±2.6 aA (51.3±9.1 aA)	19.9±1.8 aA (43.8±3.9 abA)	29.8±2.7 bA (55.0±5.1 aA)	35.2±5.0 aA (64.0±12.1 aA)	32.4±2.5 aA (56.5±6.7 aA)	28.1±3.5 aA (46.0±5.1 aA)	17.4±2.5 aA (49.5±7.5 aA)	17.1±4.4 aA (67.3±16.2 aA)	N/A	21.5±1.9 b (46.0±6.2 b)	26.9±1.1 bA (245.8±16.3 aA)	22.7±1.4 bA (276.3±16.9 aA)
**S1r1**	24.4±1.2 aA (45.5±3.8 aA)	10.2±3.1 aB (12.3±3.7 aB)	32.2±1.4 aA (57.0±5.6 aA)	22.0±2.9 aB (51.0±5.9 aA)	37.1±2.7 aA (61.5±6.1 aA)	40.8±4.4 aA (74.8±12.1 aA)	34.1±2.2 aA (50.8±6.8 aA)	29.8±1.2 aA (50.0±3.1 aA)	17.0±3.8 aA (47.8±11.0 aA)	14.3±4.1 aA (56.0±17.0 aA)	N/A	26.4±0.7 a (58.8±2.9 a)	30.5±0.8 aA (262.3±7.5 aA)	25.5±0.7 aB (303.5±27.4 aA)
**S3r2**	10.0±2.0 cA (17.5±3.8 cA)	4.8±0.7 aA (6.0±1.2 aB)	10.0±2.8 cA (16.8±6.2 cA)	7.5±1.8 bA (13.0±2.5 cA)	13.0±2.7 dA (19.5±3.1 bA)	15.9±1.7 bA (21.0±4.9 bA)	14.0±4.0 bcA (16.8±4.6 bA)	8.3±2.1 cA (10.0±2.1 cA)	12.7±1.9 aA (31.3±4.2 aA)	12.8±3.4 aA (41.3±9.8 aA)	N/A	6.3±0.9 d (11.0±2.0 d)	12.0±1.6 dA (101.5±8.5 cA)	8.7±0.7 cB (101.5±6.7 cA)
**Mean by harvests**	16.6±1.5 A (28.4±2.9 A)	6.1±1.1 B (7.5±1.3 B)	23.9±1.8 A (36.7±3.7 A)	14.5±2.2 B (29.5±5.3 B)	24.4±2.2 B (38.8±3.8 B)	30.2±3.1 A (48.2±7.3 A)	22.4±2.1 A (32.0±4.0 A)	19.7±2.6 A (28.9±4.8 A)	15.1±0.9 A (39.9±3.1 B)	15.1±1.9 A (52.7±7.1 A)	N/A	15.5±1.9 (31.8±4.5)	21.1±1.4 A (175.7±13.5 B)	17.5±1.9 B (198.4±25.5 A)

^a^ For each response variable, values (means of four replicates) not sharing a common letter, lower case (e.g. a,b) in the vertical columns for each plant part within every harvest and upper case (e.g. A,B) in the horizontal lines for each plant part between harvests (D_50_, D_65_), differ significantly (P<0.05) from each other (DMRT).

N/A = Not available before anthesis.

Inoculation with *B*. *pumilus* S1r1 recorded significantly highest % Ndfa and fixed N content in tassel (D_50_, 24.4% Ndfa or 46 mg N fixed plant^−1^), young leaves (D_50_, 32.2% Ndfa or 57 mg N fixed plant^−1^; D_65_, 22.0% Ndfa or 51 mg N fixed plant^−1^), ear leaves (D_50_, 37.1% Ndfa or 62 mg N fixed plant^−1^; D_65_, 40.8% Ndfa or 75 mg N fixed plant^−1^), old leaves (D_50_, 34.1% Ndfa or 51 mg N fixed plant^−1^; D_65_, 29.8% Ndfa or 50 mg N fixed plant^−1^) and ear (26.4% Ndfa or 59 mg N fixed plant^−1^). Meanwhile, inoculation with *K*. *pneumoniae* Fr1 produced similar results in young leaves (D_50_, 29.4% Ndfa or 51 mg N fixed plant^−1^; D_65_, 19.9% Ndfa or 44 mg N fixed plant^−1^), ear leaves (D_50_, 29.8% or 55 mg N fixed plant^−1^; D_65_, 35.2% Ndfa or 64 mg N fixed plant^−1^) and old leaves (D_50_, 32.4% Ndfa or 57 mg N fixed plant^−1^; D_65_, 28.1% Ndfa or 46 mg N fixed plant^−1^).

Among the plant parts, the highest % Ndfa was found in young leaves (10.0–32.2% Ndfa) and ear leaves (16.0–40.8% Ndfa) at D_50_ and D_65_, respectively. The lowest % Ndfa and amount of fixed N at D_65_ were found in tassel (3.3–10.2% Ndfa or 4–12 mg N fixed plant^−1^). The highest amount of fixed N was found in plant stalk at D_50_ (31–50 mg N fixed plant^−1^) and D_65_ (41–67 mg N fixed plant^−1^), albeit not the highest in % Ndfa (12.7–18.0% Ndfa). Interestingly, the maize plants inoculated with *B*. *pumilus* S1r1 had most of the fixed N concentrated in the ear leaves (62–75 mg N fixed plant^−1^), whereas other inoculated maize plants had most of their fixed N concentrated in the stalk. In tassel, % Ndfa and fixed N were significantly lower at D_65_ (6.1% Ndfa or 7 mg N fixed plant^−1^) compared to D_50_ (16.6% Ndfa or 28 mg N fixed plant^−1^). A similar trend was also observed in young leaves at D_65_, whereby % Ndfa (14.5%) and fixed N (29 mg N fixed plant^−1^) were significantly lower compared to D_50_ (23.9% Ndfa, 37 mg N fixed plant^−1^). In contrast, the ear leaves had significantly higher % Ndfa and fixed N in D_65_ than in D_50_. The amount of fixed N in the plant stalk significantly increased by 32.0% at D_65_ compared to D_50_, despite the fact that % Ndfa remained unchanged.

### Interactions between plant and bacterial parameters and time of harvests

In the present study, the soil bacterial population was found to be positively correlated with the bacterial populations in rhizosphere and root-endosphere (Table 7). The IAA production of PGPR strains was positively correlated with all the plant parameters, except in the top and root biomass at D_65_. There were significant interactions between PGPR strains and time of harvests for the parameters on total N uptake (tassel and plant top) and at. % ^15^N_e_ (tassel, young leaves and plant top) (S1 and S2 Tables).

**Table 7:** Pearson’s correlation coefficients for the plant and bacterial parameters at D_50_ (before anthesis) and D_65_ (ear harvest).

	**Pop. size in soil**	**Pop. size in rhizo.**	**Pop. size in endo.**	**IAA**	**Plant top biomass (D_50_)**	**Root biomass (D_50_)**	**Plant top biomass (D_65_)**	**Root biomass (D_65_)**
**Pop. size in soil**	1.000							
**Pop. size in rhizo.**	0.581**	1.000						
**Pop. size in endo.**	0.409*	0.035	1.000					
**IAA**	-0.696**	-0.763**	0.162	1.000				
**Plant top biomass (D**_**50**_**)**	0.261	0.471*	0.072	0.639*	1.000			
**Root biomass (D**_**50**_**)**	0.209	0.294	0.302	0.623*	0.594**	1.000		
**Plant top biomass (D**_**65**_**)**	0.112	0.255	-0.109	0.480	0.788**	0.639**	1.000	
**Root biomass (D**_**65**_**)**	0.209	0.382	0.021	-0.220	0.489*	0.566**	0.604**	1.000

Levels of significance:

*p<0.05,

**p<0.01.

n = 24.

## Discussion

In the present study, four PGPR strains (*Klebsiella* sp. Br1, *K*. *pneumoniae* Fr1, *B*. *pumilus* S1r1 and *Acinetobacter* sp. S3r2) isolated from maize roots and a reference strain (*B*. *subtilis* UPMB10) from oil palm roots were used as inoculants for maize plants grown under greenhouse conditions. According to the 16S rDNA sequence analysis, these PGPR strains belonged to: (i) *Gamma-proteobacteria*: *Klebsiella* spp. (Br1 and Fr1) and *Acinetobacter* spp. (S3r2); and (ii) *Firmicutes*: *Bacillus* spp. (UPMB10 and S1r1). Similarly, Montañez et al. [6] reported that most of their isolated bacterial genera from maize belonged to *gamma-proteobacteria* subdivision and indicated a selective maize plant association with some of these bacterial genera such as *Acinetobacter* and *Klebsiella*. Other researchers have also observed that bacteria from *Klebsiella* genus were commonly sighted near the maize root system and soil environment [8, 33].

Bacterial genera such as *Acetobacter*, *Pseudomonas*, *Azospirillum*, *Azotobacter*, *Burkholderia*, *Herbaspirillum* and *Rhizobium* have also been reported as effective maize PGPR. Inoculation with *Azotobacter* and *Azospirillum* on field-grown maize significantly increased the plant biomass by 30.7% [10]. Similarly, the co-inoculation of *Bacillus megaterium*, *Azotobacter chroococcum* and *Bacillus mucilaginous* significantly increased maize biomass and height equivalent to half of the chemical fertiliser inputs [34]. Many similar effective N_2_-fixing PGPR inoculation results have been reported on maize plant under low fertiliser-N (ca. 48 kg N ha^−1^) condition, with strains such as *Bacillus* spp., *Klebsiella* spp., *Azospirillum* spp., *Azotobacter* spp. and *Pantoea* spp. [6, 34]. These researchers attributed the increase in plant-N uptake and dry biomass of inoculated plants to PGP abilities such as BNF, phosphate solubilisation and root promoting phytohormone production namely IAA, cytokinin and gibberellin.

The five selected PGPR strains showed positive reactions on Nfb media, a differentiating media for screening PGPR with potential BNF ability [34]. The colour change phenomenon from green (pH 7.0) to blue (above pH 7.6) is due to the bromothymol blue content which changes colour with increase in pH above neutral. This increase in pH is due to the formation of fixed-ammonia from atmospheric N_2_ through natural N_2_ fixation phenomenon. In addition, this N_2_ fixation screening method allows selection of strains with higher survival traits in N-deprived soil condition [10]. Besides N_2_ fixation, these five strains have other PGP abilities, namely phosphate solubilisation and IAA production. The clear halo zones exhibited around the bacterial colonies in Pikovskaya media were in response to the solubilisation of insoluble inorganic phosphates by organic acids. Song et al. [35] attributed the efficiency to solubilise insoluble phosphates in *Burkholderia cepacia*, a PGPR isolated from cultivated soils in Korea, to its high level of organic acid productions namely gluconic acid. Other organisms such as *Pseudomonas aeruginosa* and *B*. *megaterium* have also been reported to have high phosphate solubilisation efficiency [36].

The PGPR strains in this study produced considerable amounts of IAA (5–13 μg mL^−1^) which were comparable to those presented in other reports. Among other, Egamberdiyeva et al. [37] reported 0.3 μg mL^−1^ and 0.5 μg mL^−1^ for *Pseudomonas alcaligenes* and *Mycobacterium phlei*, whereas Sachdev et al. [38] reported the highest level of 27.5 μg mL^−1^ from their collections of *K*. *pneumoniae* strains. In nature, IAA is synthesised by plants and PGPR from amino acid tryptophan, a common precursor in root exudates through transamination and decarboxylation biochemical reactions [19]. It is estimated that 80% of soil rhizosphere bacteria can produce IAA, whereas almost all (98%) the PGPR strains isolated by Arruda et al. [7] from the plant rhizospheres were able to produce IAA. In this study, the positive correlations between IAA productions by PGPR and most maize plant growth parameters (plant top and root biomass) at D_50_ strongly suggested the influence of phytohormone IAA on plant growth. However, these positive plant growth effects from IAA on plant top and root biomass were not apparent at ear harvest (D_65_), possibly due to the pot environment. IAA enhances plant growth mainly through extension of root system to reach larger soil volume for increased water and nutrient uptake. The common soil volume in all treatments could gradually limit this beneficial effects of IAA on root development, especially at the later stage of plant growth, ear harvest (D_65_) [39].

Rhizosphere usually has higher bacterial biomass and activity than the bulk soil due to the photosynthetically assimilated carbon sources such as carbohydrates, amino acids, amides, vitamins and organic acids in the root exudates [5]. This phenomenon is known as the “rhizosphere effect”, where essential nutrients for soil bacterial growth are abundantly available [40]. In this study, although the PGPR inoculated rhizospheres had higher bacterial colonisation than the uninoculated control, their differences were not statistically significant, which was possibly due to the nature of unsterilised soil condition that allowed competition between the introduced PGPR and indigenous soil bacteria [5]. In general, the total bacterial populations (10^8^ cfu g^−1^) in the PGPR inoculated rhizospheres were deemed as sufficient to promote nitrogenase activity [34, 41] in their free living state [40]. The significant correlation between bacterial populations in rhizosphere and soil suggests the phenomenon of bacterial migration from the bulk soil to the rhizosphere due to the bacterial ability to respond chemotactically [42]. Furthermore, it was hypothesised that bacterial mobility could be accelerated by soil water movement [43] in which the soil was maintained at field capacity throughout the study. Similarly, the root-endosphere of maize inoculated with PGPR showed higher bacterial populations, particularly with *Acinetobacter* sp. S3r2 treatment.

Generally, PGPR inoculations (*B*. *subtilis* UPMB10, *Klebsiella* sp. Br1, *K*. *pneumoniae* Fr1, *B*. *pumilus* S1r1 and *Acinetobacter* sp. S3r2) significantly increased the total N content and dry biomass of maize throughout the study (D_50_ and D_65_). Among the PGPR strains, inoculation with *B*. *pumilus* S1r1 and *K*. *pneumoniae* Fr1 recorded higher amounts of total N content and dry biomass in the respective whole plants, plant tops and different plant parts (tassel, young, ear and old leaves, stalk, ear and root) of maize prior to anthesis (D_50_) and ear harvest (D_65_). These increments were strongly attributed to the inherent BNF abilities, as indicated by the significantly lower at. % ^15^N_e_ in the plant tops and different plant parts of the inoculated maize compared to the uninoculated control. Lower at. % ^15^N_e_ value indicates a marked increase in percentage of N within inoculated maize plant is derived from atmospheric-N [44]. Notably, maize plants in this study were grown under unsterilised condition to simulate the actual field environment where the agricultural soil is generally unsterilised. This practice is prone to underestimate the N_2_ fixation rate of PGPR since the rate is calculated based on at. % ^15^N_e_ of reference plant, which will be indiscriminately influenced by the BNF of indigenous soil bacteria. Therefore, a suitable reference plant with similar plant type, root system and plant growth is crucial in a ^15^N isotope dilution study to minimise plant variations due to the influence of indigenous soil bacteria [45]. Factoring the conditions into account, the ^15^N isotope dilution technique is widely regarded as the most accurate and the only direct method available to quantify N status in plant and soil studies for short and long term experiments without any isotope effect or health risk under growth chamber, greenhouse and field conditions [9, 46, 47].

In the present study, maize inoculated with *B*. *pumilus* S1r1 accorded the lowest at. % ^15^N_e_ or the highest % Ndfa (as well as relative N_2_ fixed amount) in the plant top, followed by *K*. *pneumoniae* Fr1. Inoculation with *B*. *pumilus* S1r1 contributed substantial amounts of fixed N to maize plant top of 262 mg N_2_ fixed plant^−1^ (30.5% Ndfa) and 304 mg N_2_ fixed plant^−1^ (25.5% Ndfa) at D_50_ and D_65_, respectively, which were equivalent to 14.0 kg N ha^−1^ and 16.2 kg N ha^−1^, respectively, based on an equivalent planting density of 53,333 plants ha^−1^. According to Schröder et al. [48], the initial three weeks of maize plant growth requires approximately 0.50 kg N ha^−1^ day^−1^; thus, the amount of fixed N (0.25–0.28 kg N ha^−1^ day^−1^) from *B*. *pumilus* S1r1 inoculation could potentially complement ca. 50% of the total plant-N requirement. Other researchers have reported contributions of fixed N up to 26.7 kg N ha^−1^ or 70% of total plant-N in sugarcane inoculated with *Azospirillum* spp. [11]. The main advantage of N derived from BNF is due to the complete uptake of readily fixed ammonia within the plant with no losses to the environment. N losses between 50–70% of the inorganic fertiliser-N in soils through natural processes such as volatilisation, denitrification and nutrient leaching have been reported [4]. Moreover, plants cultivated in the tropical soils of low pH favour the uptake of N from ammonium or amino acid sources [49]. These N sources can influence the plant-N content since plants grown under ammonium-N condition will have twice the amount of N in their vegetative parts compared to those grown under nitrate-N condition [50].

Meanwhile, the at. % ^15^N_e_ in the plant tops and in the different plant parts of maize generally decreased upon ear harvest (D_65_). This phenomenon suggests a continuous contribution of unlabelled N from BNF and soil sources towards the dilution of at. % ^15^N_e_ in maize (inoculated and uninoculated control) until ear harvest. Nonetheless, the estimated rate of N_2_ fixed plant^−1^ day^−1^ in inoculated maize had declined after anthesis (from 3.5 to 3.1 mg N_2_ fixed plant^−1^ day^−1^), and this was possibly due to the occurrence of N feedback, as reported in *Arabidopsis* [51]. N feedback occurs when a strong N sink such as seed rapidly develops and induces high N remobilisation from senescing plant parts to phloem [46]. This sudden N spike in plant phloem has been reportedly to inhibit nitrogenase activity [51]. According to Thomas and Smart [52], this phenomenon can be mediated by effective post-anthesis plant nitrogen use efficiency (NUE) and N remobilisation to delay plant senescence.

The ^15^N isotope dilution technique can directly label the N in different plant parts to denote the two simultaneous N fluxes: (i) N remobilisation from the plant parts to grain and (ii) exogenous N uptake to the plant parts and grain [47, 53]. Conventionally, plant-N remobilisation is estimated using the differences in N contents from respective plant parts and stages of plant developments, namely stalk elongation, prior to anthesis, grain filling and maturation [54, 55, 56]. This approach is known as balance remobilisation technique which can lead to a biased estimation of remobilised N, as exogenous N uptake distribution is neglected and assumed to be completely allocated to the grain [47]. According to Masclaux-Daubresse et al. [53], the plant grain yield is determined by the collaborate efficiency of N uptake, assimilation, translocation and remobilisation. Most researchers stated that around 45–90% of N in grain of maize is derived from existing N stored in the plant prior to anthesis, while the balance is derived from post-anthesis N uptake, depending on plant genotypes and environmental conditions [14]. In this study, the majority (70–81%) of N content in maize plant top was accumulated prior to anthesis (D_50_). These stored N is continuously remobilised from structural compounds of senescing plant parts through proteolysis to developing plant parts in order to recycle the N and increase plant NUE [46]. Proteolysis is a controlled and coordinated degradation of photosynthetic protein of plastids such as chloroplast into soluble proteins for N remobilisation [53].

N remobilisation in plants could influence C production, as remobilised N is primarily sourced and initiated from senescing photosynthetic plant parts [57]. According to Uhart and Andrade [13], certain soil conditions such as limited rate of N fertilisation and water supply could increase N remobilisation efficiency in plants but would result in reduced C filling and grain yield. Conversely, delayed plant senescence can increase C filling and grain yield, but it will lead to decreased grain protein content [53]. Consequently, PGPR applications could provide a solution to manipulate plant senescence and continuous exogenous fixed N to plants, and concomitantly maintain high grain yield and protein content [52]. This pioneering study has demonstrated the effects of PGPR on N fluxes in maize plant prior to anthesis (D_50_) and at ear harvest (D_65_) which could create a new approach to plant-N management.

Prior to anthesis (D_50_), the majority of N assimilated during the early growth of maize was derived from labelled fertiliser-urea source, instead of unlabelled soil and atmospheric sources, as indicated by the highest at. % ^15^N_e_ in the old leaves of maize (D_50_ and D_65_). This phenomenon demonstrated the role of old leaves as a major sink for N during the early maize growth [57]. Concurrently, N remobilisation from old leaves to photosynthetically active plant parts such as ear and young leaves might have occurred before anthesis, as suggested by the lowest N uptake in the old leaves (D_50_) and the significant increments in N uptake and dry biomass of young leaves at D_65_. The old leaves continued to be the source of remobilised N for developing plant parts (young leaves, stalk and ear) after anthesis, as indicated in the significantly lower at. % ^15^N_e_ value at D_65_, while the N uptake and dry biomass parameters remained similarly low at D_50_. As ear leaves were optimal for photosynthate production at D_50_, the N usage for further organ growth has significantly reduced, as exhibited in the unchanged N uptake and dry biomass parameters of ear leaves between D_65_ and D_50_ harvests.

At D_50_, some tassels have experienced dehiscence, particularly from the uninoculated and *Klebsiella* sp. Br1 treated maize plants, as exhibited in their unchanged N uptakes at D_65_ harvest. At the same time, inoculations with other superior N_2_-fixing PGPR (*B*. *pumilus* S1r1, *K*. *pneumoniae* Fr1, *B*. *subtilis* UPMB10 and *Acinetobacter* sp. S3r2) have delayed the dehiscence of tassels, thus exhibiting significant reductions in N uptakes at D_65_ harvest. In addition, the influence of PGPR on N remobilisation in maize was clearly demonstrated in the significant interactions found between the PGPR strains and the time of harvests in N uptake and at. % ^15^N_e_ parameters of tassel.

During grain formation, the ear leaves functioned as supporting organs to continuously channel exogenous and remobilised N to ears [55]. However, the N remobilisation in ear leaves determined using balance remobilisation technique (N uptake and dry biomass) showed no apparent changes between D_50_ and D_65_. Favourably, the significantly lower at. % ^15^N_e_ in ear leaves of D_65_ harvest suggested that the N content was diluted with “later” unlabelled-N uptakes from BNF and soil sources. The significant increments of fixed N (% Ndfa) in ear leaves of inoculated maize at D_65_ further emphasised the role of ear leaves as temporary transits to ears, which had the lowest at. % ^15^N_e_ at D_65_. Meanwhile, *B*. *pumilus* S1r1 displayed its superiority in BNF as indicated by the highest amount of fixed N found in the ear leaves instead of the plant stalk, where most fixed N of other PGPR strains was located at D_65_ harvest.

Amidst the fluctuations of N fluxes in the tassel, leaves (young, ear and old) and ear of maize, the at. % ^15^N_e_ in plant stalk remained mostly consistent throughout the plant growth, despite having the highest N uptake and dry biomass at D_50_ and D_65_ harvests. In addition to the significant increments of plant biomass and N uptake of stalk at D_65_, these phenomena demonstrated the role of plant stalk as an important N reservoir for maize [54, 55]. According to Ta and Weiland [57], plant stalks could contribute similar amounts of remobilised N as the accumulated N in plant leaves to the grain, ca. 40%. The present plant stalks which contained between 30.5–31.3% of total plant top N could possibly have several times higher nitrate concentration than the N in the leaves [50] and the nitrate-N is readily available for N remobilisation [54]. In general, the present study has demonstrated that leaves (old, ear and young), tassel and stalk of maize plants had served successively as N sinks and N sources towards ear formation.

Nonetheless, plant roots were not investigated in the present study using ^15^N isotope dilution technique due to their lesser involvement in N fluxes, as they exhibited a low representation of plant biomass (<8%) compared to above ground plant parts [54]. According to Salon et al., [46], plant roots function mostly as mechanical support and nutrient uptake for growth. Furthermore, the at. % ^15^N_e_ in the above and below ground plant parts are normally similar and either of these plant parts will give similar % Ndfa [31]. This study has clearly demonstrated that the isolated PGPR, particularly *B*. *pumilus* S1r1 which fixed a significant amount of atmospheric N_2_, promoted vegetative growth and delayed plant senescence of maize, thereby produced a higher N content and yield of maize ear.

## Conclusions

This greenhouse study has demonstrated that inoculation with locally isolated PGPR strains, mainly *Bacillus pumilus* S1r1, *Klebsiella pneumoniae* Fr1, *Bacillus subtilis* UPMB10 and *Acinetobacter* sp. S3r2 could significantly increase plant-N uptake, dry biomass and ear yield of maize. These increments are mainly attributed to the BNF ability of the strains, namely *B*. *pumilus* S1r1, which is able to fix up to 304 mg N_2_ fixed plant^−1^ at ear harvest (D_65_), and possibly other PGP abilities such as IAA production and phosphate solubilisation. The biomass production of plant tops and roots is correlated positively with IAA production by the PGPR strains. The maize plants inoculated with PGPR strains also exhibited delayed plant senescence, particularly in the tassel, which consequently improved the ear yield. This positively demonstrates that the remobilisation of N accumulated in maize top prior to anthesis from the leaves (old, ear and young), tassel and stalk have served successively as N sinks and N sources toward the ear yield. Thus, this study indicates that PGPR inoculation can be considered as an alternative technique to improve grain yield besides the conventional plant breeding method for “delayed senescence” varieties, which is time consuming and tedious. Further studies are necessary to evaluate (i) the suitability and performance of *B*. *pumilus* S1r1 on maize under field conditions and (ii) the PGPR mechanisms involved in delaying plant senescence for higher N accumulation.

## Supporting Information

S1 Fig Effects of preliminary PGPR inoculation on dry weight of maize top. Asterisk,* on a bar indicates significant difference by Dunnett’s test (Uninoculated 1/3 N control) at p<0.05. Error bar indicates standard errors.
(PDF)

S2 Fig Effects of preliminary PGPR inoculation on total N uptake of maize. Asterisk,* on a bar indicates significant difference by Dunnett’s test (Uninoculated 1/3 N control) at p<0.05. Error bar indicates standard errors.
(PDF)

S3 Fig Red-gel stained 1% agarose gel displaying amplified DNA products under UV-transilluminator. Lanes: 1, Fr1 DNA; 2, S1r1 DNA; 3, S3r2 DNA; 4, Br1 DNA; 5, UPMB10 DNA; M, 1kb DNA ladder (Fermentas GeneRulerTM).
(PDF)

S1 Table ANOVA Output of total N uptake in plant top and in different parts of maize inoculated with PGPR at D50 and D65 harvests. (PDF)

S2 Table ANOVA Output of at. % 15Ne in plant top and in different parts of maize inoculated with PGPR at D50 and D65 harvests. (PDF)

## References

[1] Nor HMS, Faridah H, Sharizan A, Sebrina SS. Jagung manis hybrid baru Hibrimas. Buletin Teknologi MARDI; 2012.

[2] Ministry of Agriculture and Agro-Based Industry Malaysia. Agrofood statistics 2013. Malaysia; 2014.

[3] Dierolf T, Fairhurst T, Mutert E. Soil fertility kit: a toolkit for acid, upland soil fertility management in Southeast Asia. Potash & Phosphate Institute (PPI) Singapore; 2001.

[4] Hodge A, Robinson D, Fitter A. Are microorganisms more effective than plants at competing for nitrogen? Trends Plant Sci. 2000; 5: 304–308. 10871903 10.1016/s1360-1385(00)01656-3

[5] Lugtenberg B, Kamilova F. Plant-growth-promoting rhizobacteria. Annu. Rev. Microbiol. 2009; 63: 541–556. 10.1146/annurev.micro.62.081307.162918 19575558

[6] Montañez A, Abreu C, Gill PR, Hardarson G, Siracdi M. Biological nitrogen fixation in maize (*Zea mays* L.) by 15N isotope-dilution and identification of associated culturable diazotrophs. Biol. Fertil. Soils 2009; 45: 253–263.

[7] Arruda L, Beneduzi A, Martins A, Lisboa B, Lopes C, Bertolo F, et al Screening of rhizobacteria from maize (*Zea mays* L.) in Rio Grande do Sul State (South Brazil) and analysis of their potential to improve plant growth. Appl. Soil Ecol. 2013; 63: 15–22.

[8] Chelius MK, Triplett EW. The diversity of archaea and bacteria in association with the roots of *Zea mays* L. Microb. Ecol. 2001; 41: 252–263. 11391463 10.1007/s002480000087

[9] Zakry FAA, Shamsuddin ZH, Rahim KA, Zakaria ZZ, Rahim AA. Inoculation of *Bacillus sphaericus* UPMB-10 to young oil palm and measurement of its uptake of fixed nitrogen using the ^15^N isotope dilution technique. Microbes Environ. 2012; 27(3): 257–262. 22446306 10.1264/jsme2.ME11309 PMC4036051

[10] Piromyou P, Buranabanyat B, Tantasawat P, Tittabutr P, Boonkerd N, Teaumroong N. Effect of plant growth promoting rhizobacteria (PGPR) inoculation on microbial community structure in rhizosphere of forage corn cultivated in Thailand. Eur. J. Soil Biol. 2011; 47: 44–54.

[11] Boddey RM, Oliveira OC, Urquiga S, Reis VM, Olivares FL, Baldani VLD, et al Biological nitrogen fixation associated with sugar cane and rice: contributions and prospects for improvement. Plant Soil 1995; 174: 195–209.

[12] Matiru VN, Dakora FD. Potential use of rhizobial bacteria as promoters of plant growth for increased yield in landraces of African cereal crops. Afr. J. Biotechnol. 2004; 3: 1–7.

[13] Uhart SA, Andrade FH. Nitrogen and carbon accumulation and remobilisation during grain filling in maize under different source/sink ratios. Crop Sci. 1995; 35: 183–190.

[14] Kichey T, Hirel B, Heumez E, Dubois F, Le Gouis J. In winter wheat (*Triticum aestivum* L.), post-anthesis nitrogen uptake and remobilisation to the grain correlates with agronomic traits and nitrogen physiological markers. Field Crop. Res. 2007; 102: 22–32.

[15] Hoben HJ, Somasegaran P. Comparison of the pour, spread, and drop plate methods for enumeration of *Rhizobium* spp. in inoculants made from presterilized peat. Appl. Environ. Microbiol. 1982; 44: 1246–1247. 16346141 10.1128/aem.44.5.1246-1247.1982 PMC242176

[16] Shamsuddin ZH, Roslina AW. Isolation of *Azospirillum* spp. from oil palm roots The proceedings of the 18th Microbiology Symposium, Malaysian Society for Microbiology, Kuching, Sarawak; 1995 pp. 1–9.

[17] Baldani VLD, Döbereiner J. Host-plant specificity in the infection of cereals with *Azospirillum* spp. Soil Biol. Biochem. 1980; 12: 433–439.

[18] Pikovskaya RI. Mobilization of phosphorus in soil in connection with vital activity of some microbial species. Microbiologia 1948; 17: 362–370.

[19] Glickmann E, Dessaux Y. A critical examination of the specificity of the Salkowski reagent for indolic compounds produced by phytopathogenic bacteria. Appl. Environ. Microbiol. 1995; 16: 793–796. 10.1128/aem.61.2.793-796.1995 PMC1388360 16534942

[20] Koh MC, Lim CH, Karichiappan K, Chua SB, Chew ST, Phang STW. Random amplified polymorphic DNA fingerprints for identification of red meat species. Meat Sci. 1998; 48: 275–285. 22063076 10.1016/s0309-1740(97)00104-6

[21] Weisburg WG, Bams SM, Pelletier DA, Lane DJ. 16S ribosomal DNA amplication for phylogenetic study. J. Bacteriol. 1991; 173: 697–703. 1987160 10.1128/jb.173.2.697-703.1991 PMC207061

[22] Edgar RC. Muscle: multiple sequence alignment with high accuracy and high throughput. Nucleic Acids Res. 2004; 32:1792–1797. 15034147 10.1093/nar/gkh340 PMC390337

[23] Tamura K, Peterson D, Peterson N, Stecher G, Nei M, Kumar S. MEGA5: molecular evolutionary genetics analysis using maximum likelihood, evolutionary distance, and maximum parsimony method. Mol. Biol. Evol. 2011; 28: 2731–2739. 10.1093/molbev/msr121 21546353 PMC3203626

[24] Hall BG. Building phylogenetic trees from molecular data with MEGA. Mol. Biol. Evol. 2013; 5: 1229–1235. 10.1093/molbev/mst012 23486614

[25] Bremner JM. Nitrogen total In: Spark DL, editor. Methods of soil analysis, part 3: chemical methods. Wisconsin, USA Soil Science Society of America; 1996: pp. 1085–1121.

[26] Wang DL, Anderson DW. Direct measurement of organic carbon content in soils by Leco CR-12 carbon analyser. Commun. Soil Sci. Plan. 1998; 29: 15–21.

[27] Bray RH, Kurtz LT. Determination of total, organic and available form of phosphorus in soils. Soil Sci. 1945; 59: 39–45.

[28] Thomas GW. Exchangeable cations In: Page AL, Miller RH, Keeny DR, editors. Methods of soil analysis, part 2: chemical and microbiological properties. Agronomy monograph no. 9 (2nd Edition). Madison, USA American Society of Agronomy; 1982 pp. 159–165.

[29] Richards LA, Fireman M. Pressure-plate apparatus for measuring moisture sorption and transmission by soils. Soil Sci. 1943; 56: 395–404.

[30] Malaysian Agricultural Research and Development Institute (MARDI). Anggaran kos pengeluaran dan pendapatan bagi tanaman kontan, Kementerian Pertanian dan Industri Asas Tani. Malaysia; 2006.

[31] International Atomic Energy Agency (IAEA). Use of isotope and radiation methods in soil and water management and crop nutrition. IAEA-TCS-14. Vienna, Austria; 2001.

[32] Stell RGD, Torrie JH, Dickey DA. Principles and procedures of statistics: a biometrical approach. McGraw-Hill, New York; 1980.

[33] Mehnaz S, Kowalik T, Reynolds B, Lazarovitz G. Growth promoting effects of corn (*Zea mays*) bacterial isolates under greenhouse and field conditions. Soil Biol. Biochem. 2010; 42: 1848–1856.

[34] Wu SC, Cao ZH, Li ZG, Cheung KC, Wong MH. Effects of biofertilizer containing N-fixer, P and K solubilizers and AM fungi on maize growth: a greenhouse trial. Geoderma 2005; 125: 155–166.

[35] Song OR, Lee SJ, Lee YS, Lee SC, Kim KK, Choi YL. Solubilization of insoluble inorganic phosphate by *Burkholderia cepacia* DA23 isolated from cultivated soil. Braz. J. Microbiol. 2008; 39: 151–156. 10.1590/S1517-838220080001000030 24031195 PMC3768359

[36] Yadav J, Verma JP. Effect of seed inoculation with indigenous *Rhizobium* and plant growth promoting rhizobacteria on nutrients uptake and yields of chickpea (*Cicer arietinum* L.). Eur. J. Soil Biol. 2014; 63: 70–77.

[37] Egamberdiyeva D. The effect of plant growth promoting bacteria on growth and nutrient uptake of maize in two different soils. Appl. Soil Ecol. 2007; 36: 184–189.

[38] Sachdev DP, Chaudhari HG, Kasture VM, Dhavale DD, Chopade BA. Isolation and characterization of indole acetic acid (IAA) producing *Klebsiella pneumonia* strains from rhizosphere of wheat (*Triticum aestivum*) and their effect on plant growth. Indian J. Exp. Biol. 2009; 47: 993–1000. 20329704

[39] Wang B, Seiler JR, Mei C. *Burkholderia phytofirmas* strain PsJN advanced development and altered leaf level physiology of switchgrass. Biomass Bioenerg. 2015; 83: 493–500. 10.1016/j.plaphy.2014.11.008 25461696

[40] Bürgmann H, Stefan M, Micheal B, Franco W, Josef Z. Effects of model root exudates on structure and activity of a soil diazotroph community. Environ. Microbiol. 2005; 7: 1711–1724. 16232286 10.1111/j.1462-2920.2005.00818.x

[41] James EK. Nitrogen fixation in endophytic and associative symbiosis. Field Crop. Res. 2000; 65: 197–209.

[42] Compant S, Clément C, Sessitsch A. Plant growth-promoting bacteria in the rhizo- and endosphere of plants: their role, colonization, mechanisms involved and prospects for utilization. Soil Biol. Biochem. 2010; 42: 669–678.

[43] Benizri E, Baudoin E, Guckert A. Root colonization by inoculated plant growth rhizobacteria. Biocontrol Sci. Techn. 2001; 11: 557–574.

[44] Boddey RM. Methods for quantification of nitrogen fixation associated with gramineae. CRC Crit. Rev. Plant Sci. 1987; 6: 209–266.

[45] Danso SKA, Hardarson G, Zapata F. Misconceptions and practical problems in the use of 15N soil enrichment techniques for estimating N2 fixation. Plant Soil 1993; 152: 25–52.

[46] Salon C, Munier-Jolain NG, Duc G, Viosin A, Grandgirard D, Larmurec A, et al Grain legume seed filling in relation to nitrogen acquisition: a review and prospects with particular reference to pea. Agronomie 2001; 21: 539–552.

[47] Gallais A, Coque M, Le Gouis J, Prioul JL, Hirel B, Quilléré I. Estimating the proportion of nitrogen remobilization and of postsilking nitrogen uptake allocated to maize kernels by nitogen-15 labeling. Crop Sci. 2007; 47: 685–693.

[48] Schröder JJ, Neeteson JJ, Oenema O, Struik PC. Does the crop or the soil indicate how to save nitrogen in maize production? Reviewing the state of the art. Field Crop. Res. 2000; 66: 151–164.

[49] Maathuis F. Physiological functions of mineral macronutrients. Curr. Opin. Plant Biol. 2009; 12: 250–258. 10.1016/j.pbi.2009.04.003 19473870

[50] Barker AV, Bryson GM. Nitrogen In: Barker AV, Pilbeam DJ, editors. Handbook of plant nutrition. Florida, CRC Press, USA; 2007, pp. 21–50.

[51] Diaz C, Lemaître T, Christ A, Azzopardi M, Kato Y, Sato F, et al Nitrogen recycling and remobilization are differentially controlled by leaf senescence and development stage in *Arabidopsis* under low nitrogen nutrition. Plant Physiol. 2008; 147: 1437–1449. 10.1104/pp.108.119040 18467460 PMC2442554

[52] Thomas H, Smart CM. Crops that stay green. Ann. Appl. Biol. 1993; 123: 193–219.

[53] Masclaux-Daubresse C, Daniele-Vedele F, Dechorgnat J, Chardon F, Gaufichon L, Suzuki A. Nitrogen uptake, assimilation and remobilization in plants: challenges for sustainable and productive agriculture. Ann. Bot. 2010; 105: 1141–1157. 10.1093/aob/mcq028 20299346 PMC2887065

[54] Cliquet JB, Deléens E, Bousser A, Martin M, Lescure JC, Piroul JL, et al Estimation of the carbon and nitrogen allocation during stalk elongation by 13C and 15N tracing in *Zea mays* L. Plant Physiol. 1990; 92: 78–87. 10.1104/pp.92.1.79 PMC1062251 16667269

[55] Cliquet JB, Deléens E, Mariotti A. C and N mobilization from stalk and leaves during kernel filling by 13C and 15N tracing in *Zea mays* L. Plant Physiol. 1990; 94: 1547–1553. 16667888 10.1104/pp.94.4.1547 PMC1077419

[56] Masclaux-Daubresse C, Reisdorf-Cren M, Orsel M. Leaf nitrogen remobilisation for plant development and grain filling. Plant Biol. 2008; 10: 23–36. 10.1111/j.1438-8677.2008.00097.x 18721309

[57] Ta CT, Weiland RT. Nitrogen partitioning in maize during ear development. Crop Sci. 1992; 32: 443–451.

