# Supplementary material for: Plant Growth-Promoting Rhizobacteria Inoculation to Enhance Vegetative Growth, Nitrogen Fixation and Nitrogen Remobilisation of Maize under Greenhouse Conditions
Source: PLoS One. 2016 Mar 24;11(3):e0152478. doi: 10.1371/journal.pone.0152478 (PMC4807084; doi:10.1371/journal.pone.0152478)
Supplement: S2 Fig — Asterisk,* on a bar indicates significant difference by Dunnett’s test (Uninoculated 1/3 N control) at p<0.05. Error bar indicates standard errors. (PDF) [file pone.0152478.s002.pdf]

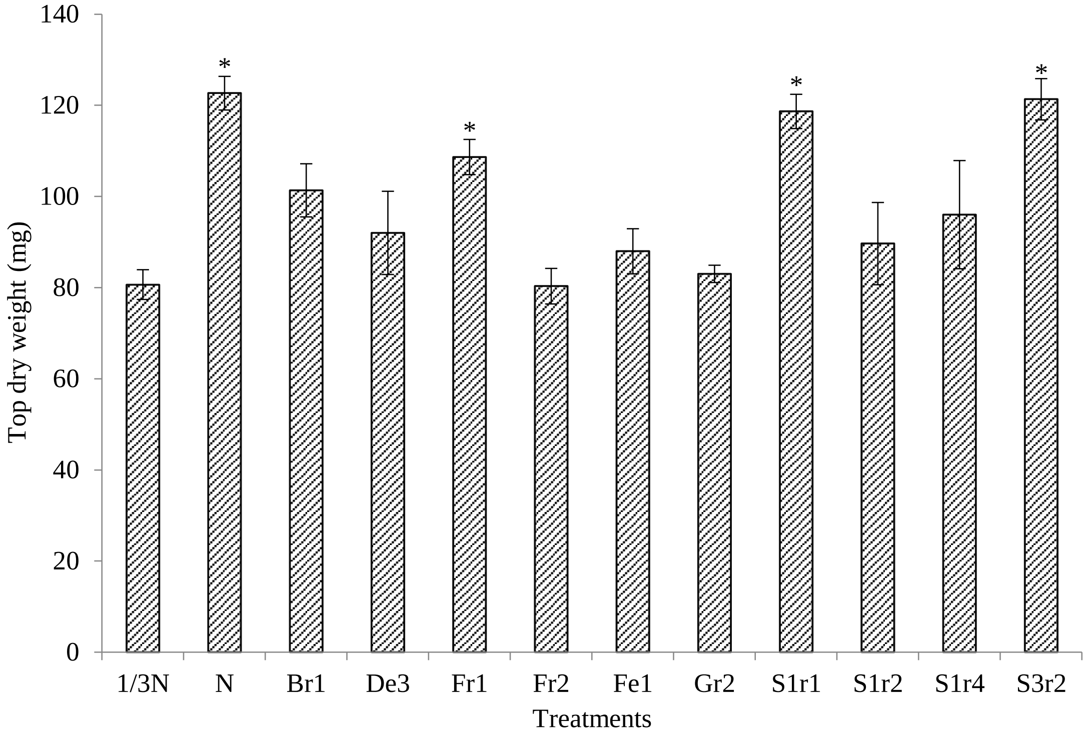

S2 Fig. Effects of preliminary PGPR inoculation on dry weight of maize top. Asterisk,* on a bar indicates significant difference by Dunnett's test (Uninoculated 1/3 N control) at $p<0.05$. Error bar indicates standard errors.
