# Supplementary material for: Plant Growth-Promoting Rhizobacteria Inoculation to Enhance Vegetative Growth, Nitrogen Fixation and Nitrogen Remobilisation of Maize under Greenhouse Conditions
Source: PLoS One. 2016 Mar 24;11(3):e0152478. doi: 10.1371/journal.pone.0152478 (PMC4807084; doi:10.1371/journal.pone.0152478)
Supplement: S3 Fig — Lanes: 1, Fr1 DNA; 2, S1r1 DNA; 3, S3r2 DNA; 4, Br1 DNA; 5, UPMB10 DNA; M, 1kb DNA ladder (Fermentas GeneRulerTM). (PDF) [file pone.0152478.s003.pdf]

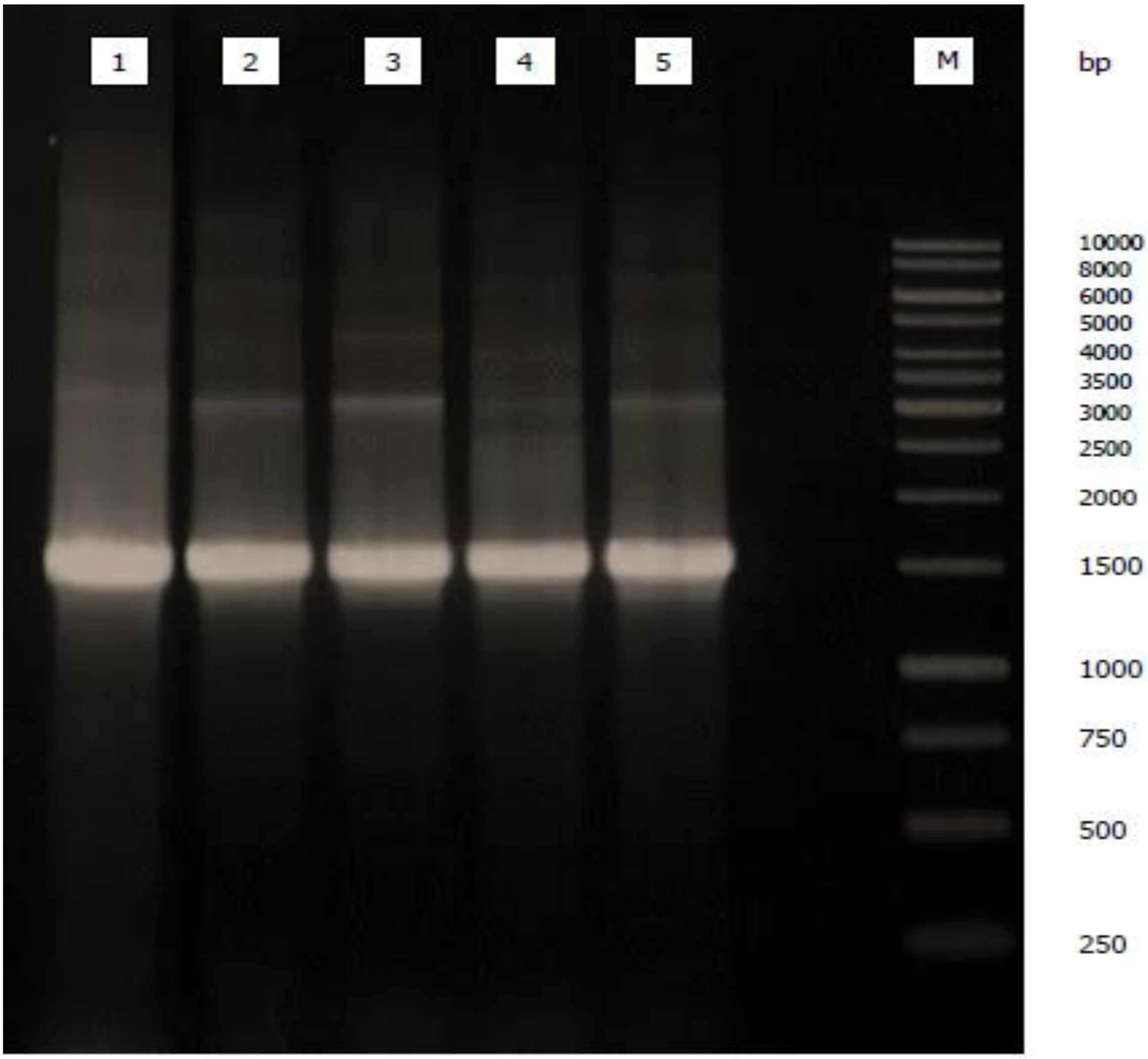

S3 Fig. Red-gel stained 1% agarose gel displaying amplified DNA products under UV-transilluminator. Lanes: 1, Fr1 DNA; 2, S1r1 DNA; 3, S3r2 DNA; 4, Br1 DNA; 5, UPMB10 DNA; M, 1kb DNA ladder (Fermentas GeneRuler^{TM}).
