# Supplementary material for: Plant Growth-Promoting Rhizobacteria Inoculation to Enhance Vegetative Growth, Nitrogen Fixation and Nitrogen Remobilisation of Maize under Greenhouse Conditions
Source: PLoS One. 2016 Mar 24;11(3):e0152478. doi: 10.1371/journal.pone.0152478 (PMC4807084; doi:10.1371/journal.pone.0152478)
Supplement: S1 Table — (PDF) [file pone.0152478.s004.pdf]

S1 Table. ANOVA Output of total N uptake in plant top and in different parts of maize inoculated with PGPR at D_{50} and D_{65} harvests.

Tassel

Source	DF	Anova SS	Mean Square	F Value	Pr> F
Block	3	7955.063	2651.688	14.11	<.0001
Treatment	5	9861.354	1972.271	10.49	<.0001
D	1	19642.52	19642.52	104.49	<.0001
Treatment*D	5	3843.354	768.6708	4.09	0.0053

Young leaves

Source	DF	Anova SS	Mean Square	F Value	Pr > F
Block	3	17925.08	5975.028	8.42	0.0003
Treatment	5	47713.92	9542.783	13.45	<.0001
D	1	21000.33	21000.33	29.61	<.0001
Treatment*D	5	2871.667	574.3333	0.81	0.5511

Ear leaves

Source	DF	Anova SS	Mean Square	F Value	Pr > F
Block	3	17455.58	5818.528	12.18	<.0001
Treatment	5	35057.92	7011.583	14.68	<.0001
D	1	645.3333	645.3333	1.35	0.2535
Treatment*D	5	3497.167	699.4333	1.46	0.2281

Old leaves

Source	DF	Anova SS	Mean Square	F Value	Pr > F
Block	3	619.8958	206.6319	0.29	0.8351
Treatment	5	38178.44	7635.688	10.57	<.0001
D	1	346.6875	346.6875	0.48	0.4933
Treatment*D	5	950.4375	190.0875	0.26	0.9299

Stalk

Source	DF	Anova SS	Mean Square	F Value	Pr > F
Block	3	5474.063	1824.688	1.93	0.1445
Treatment	5	41735.44	8347.088	8.81	<.0001
D	1	106879.7	106879.7	112.85	<.0001
Treatment*D	5	4499.438	899.8875	0.95	0.4621

Plant top

Source	DF	Anova SS	Mean Square	F Value	Pr > F
Block	3	197194.1	65731.35	22.36	<.0001
Treatment	5	951914.4	190382.9	64.75	<.0001
D	1	931582.7	931582.7	316.83	<.0001
Treatment*D	5	40739.19	8147.838	2.77	0.0338
