# Supplementary material for: Plant Growth-Promoting Rhizobacteria Inoculation to Enhance Vegetative Growth, Nitrogen Fixation and Nitrogen Remobilisation of Maize under Greenhouse Conditions
Source: PLoS One. 2016 Mar 24;11(3):e0152478. doi: 10.1371/journal.pone.0152478 (PMC4807084; doi:10.1371/journal.pone.0152478)
Supplement: S2 Table — (PDF) [file pone.0152478.s005.pdf]

S2 Table. ANOVA Output of at. % $^{15}N_e$ in plant top and in different parts of maize inoculated with PGPR at D_{50} and D_{65} harvests.

Tassel

Source	DF	Anova SS	Mean Square	F Value	Pr> F
Block	3	0.012147	0.004049	4.28	0.0117
Treatment	5	0.153147	0.030629	32.38	<.0001
D	1	0.014981	0.014981	15.84	0.0004
Treatment*D	5	0.040344	0.008069	8.53	<.0001

Young leaves

Source	DF	Anova SS	Mean Square	F Value	Pr > F
Block	3	0.012669	0.004223	1.91	0.1471
Treatment	5	0.603509	0.120702	54.61	<.0001
D	1	0.014456	0.014456	6.54	0.0153
Treatment*D	5	0.05342	0.010684	4.83	0.002

Ear leaves

Source	DF	Anova SS	Mean Square	F Value	Pr > F
Block	3	0.006801	0.002267	0.79	0.5076
Treatment	5	1.178573	0.235715	82.26	<.0001
D	1	0.333667	0.333667	116.44	<.0001
Treatment*D	5	0.027611	0.005522	1.93	0.1163

Old leaves

Source	DF	Anova SS	Mean Square	F Value	Pr > F
Block	3	0.025927	0.008642	1.73	0.1795
Treatment	5	1.430481	0.286096	57.34	<.0001
D	1	0.061992	0.061992	12.42	0.0013
Treatment*D	5	0.04049	0.008098	1.62	0.1813

Stalk

Source	DF	Anova SS	Mean Square	F Value	Pr > F
Block	3	0.012777	0.004259	1.26	0.3027
Treatment	5	0.202437	0.040487	12.02	<.0001
D	1	0.006745	0.006745	2	0.1665
Treatment*D	5	0.006196	0.001239	0.37	0.8669

Plant top

Source	DF	Anova SS	Mean Square	F Value	Pr > F
Block	3	0.000268	8.93E-05	0.24	0.8699
Treatment	5	0.614593	0.122919	326.26	<.0001
D	1	0.107352	0.107352	284.94	<.0001
Treatment*D	5	0.012611	0.002522	6.69	0.0002
